# Homesickness in asylum seekers: The role of mental health and migration-related factors

**DOI:** 10.3389/fpsyt.2022.1034370

**Published:** 2022-10-31

**Authors:** Rita Rosner, Maria Hagl, Leonie Bücheler, Hannah Comtesse

**Affiliations:** ^1^Department of Psychology, Catholic University Eichstaett-Ingolstadt, Eichstaett, Germany; ^2^Freelance Researcher, Munich, Germany

**Keywords:** homesickness, refugees, depression, posttraumatic stress, loss

## Abstract

**Background:**

While homesickness in refugees is a recurring theme in clinical practice, respective research in this population is scarce. The *Utrecht Homesickness Scale* (UHS) allows distinguishing between certain aspects of homesickness, namely genuine separation distress like missing family and friends or yearning for home on the one side and problems regarding adjustment to the new situation on the other; so far, the instrument was applied mainly in samples of university students, and never in refugees.

**Objective:**

We aimed to explore homesickness in a refugee population and its association with mental health symptoms and migration-related factors. In addition, we wanted to evaluate the UHS’s factor structure in a sample other than students.

**Methods:**

Individuals from different countries (*N* = 99) seeking asylum in Germany were assessed for homesickness, migration-related variables (e.g., number of losses and stage of the asylum proceedings), and mental health symptoms (symptoms of depression, posttraumatic stress and prolonged grief). After exploratory factor analysis, standardized mean factors scores were fed into subsequent correlational and regression analyses to identify the most prominent predictors of homesickness scores.

**Results:**

The participants showed substantial levels of homesickness. We found a three-factor solution that implied distinct factors regarding (1) adjustment difficulties and loneliness, (2) ruminations about home, and (3) missing family and/or friends. The total homesickness score was associated with mental health but regression analyses with the three mean factor scores showed differential associations with mental health and migration-related variables. While adjustment difficulties and loneliness were—besides time since arrival in Germany—associated with mental health problems (depressive and posttraumatic stress symptoms), ruminating about home was predicted by migration-related variables (number of losses and asylum status). For the factor scores regarding missing family and/or friends, the regression model was not significant.

**Conclusion:**

The assessment of homesickness in refugee populations is feasible and of clinical importance, especially when distinguishing between separation distress and difficulties with adjusting to the new situation.

## Introduction

Among all migrant groups, refugees probably encounter the most difficult circumstances [see (1)], their situation aggravated by the circumstances that forced them to leave their home, but also by poor living conditions and varying levels of support and acceptance in the host countries. Several meta-analyses attest to refugees’ mental health burden related to traumatic experiences before and during forced migration [e.g., (2–4)] which is moderated by post-migration stressors [e.g., (5); for an overview, see (6)]. One of these stressors is the simple fact of having left behind one’s home—in the case of forced displacement or political persecution often without knowing when a return will be possible, or if ever. Bhugra et al. (1) describe how migration universally leads to the “loss of the familiar” (p. 4) and that grieving for this loss can cause significant distress, a phenomenon that Eisenbruch (7) called “cultural bereavement.” Although, according to Eisenbruch, nostalgia and homesickness in “uprooted people” [(7); p. 674] have been described from early on, these concepts seldom found their way into current research on post-migration stress in refugees. Notwithstanding, when assessed with a list of 18 possible post-migration stressors, homesickness ranked first in a sample of refugees in the Netherlands (8), and in a Swiss study, nearly half of a sample of asylum seekers reported being homesick frequently (9). In a qualitative study, volunteer sponsors who supported Kosovar refugee women in Canada named homesickness and worrying about family members back in Kosovo as a common mental health concern in these women (10). In another study, separation from family members—in terms of worrying for their safety and feelings of helplessness regarding their needs but also in terms of unmet needs of emotional support and cultural continuity—not only emerged as a foremost concern in refugees resettled in the US in qualitative interviews but also as an important predictor for mental health in quantitative analysis (11). Such results correspond well with clinical experience, where these kind of worries but also simply missing home and family members or friends is a recurring theme in the treatment of refugees suffering from PTSD and/or depression and seem to interfere with improvement [e.g., (12)]. However, while research on the impact of refugees being separated from family members is increasing [e.g., (11, 13, 14)], there are few studies looking at the concept of overall homesickness. Up to now, research does not allow for conclusions whether homesickness in refugees should be considered as a risk factor that aggravates mental health symptoms or rather as a consequence of poor psychological adjustment—it could be both—or the manifestations of homesickness might simply overlap with those of depression and grief (see below).

So far, homesickness as a psychological construct assessed with a psychometrically validated instrument in adults was mostly researched in student samples as a recent systematic review showed for the last two decades (15). Earlier empirical and theoretical work also focused mostly on young people and temporary relocations [e.g., (16)], and in their review, Stroebe et al. (17) explicitly excluded studies with samples of refugees and other migrants—which makes perfectly sense in the light of the obvious differences in experiences of students leaving home to study abroad and people who were forced to leave their homes, not knowing whether they will ever see it again. Still, homesickness seems to be a rather universal experience that manifests on several levels [(18), see also (17)]: emotional (e.g., yearning for home, feeling lonely, downcast mood), cognitive (e.g., preoccupying thoughts of what is missed), behavioral (e.g., withdrawn or aggressive behavior), and somatic (e.g., weight loss, sleep disturbances). From a clinical view, severe forms of homesickness might be classified as adjustment disorder (18). There is a substantial overlap with depressive symptoms, and in addition, homesickness has been found related to anxiety and anger [e.g., (19); in a Dutch population sample]. Stroebe et al. (17) argue that homesickness *per se* is best conceptualized as a grief reaction as it occurs in consequence of separation from loved ones (albeit less irrevocable than death), and as feelings of yearning and longing predominate. In both types of separation (temporary or perpetual loss), there is a need for adapting to the new situation, but adjustment-related phenomena—or as Stroebe et al. (20) put it, the “new place factor”—should be understood as a different domain and measured separately from the loss-related “home factor.” What’s more, overly yearning for home might interfere with adjustment to a new place and—vice versa—adjustment problems might exacerbate homesickness *per se*. Stroebe et al.’s distinction could be especially fruitful in research with refugees where separation from family as well as acculturative stress have been identified as important contributing factors in poor post-migration adaptation and mental health (21–23).

To explore the association between homesickness and mental health in a refugee population, we present a secondary analysis of a sample of 99 individuals who had applied for asylum in Germany [(24), see also (25)]. Rates for a probable diagnosis of posttraumatic stress disorder (PTSD) were high (45%) as well as for depression (42%). In addition, nearly all participants (92%) reported the loss of at least one loved person and 20% fulfilled the diagnosis of prolonged grief disorder (PGD) according to the criteria proposed by Prigerson et al. (26). In the following, we present our findings on homesickness assessed with the *Utrecht Homesickness Scale* (UHS; 27), with the aim to disentangle its association with prolonged grief symptoms as well as depressive and posttraumatic stress symptoms while taking migration-related variables into account, for example number of losses and the stage of the asylum proceedings. With these exploratory analyses, we hope to shed light on the question whether homesickness should be regarded as being predominantly a grief phenomenon and whether different aspects of homesickness should be assessed separately as suggested by Stroebe et al. (20).

## Materials and methods

### Participants and procedure

Altogether 104 individuals who had applied for asylum in Germany were recruited and interviewed in five collective accommodation facilities in Bavaria, Germany. We informed potential participants via posters and mailing lists as well as by going from door to door and personally informing the respective residents with the help of two cultural mediators. In particular, we clarified that study participation was voluntary and not related to the asylum proceedings in any way, and that any information given in the study would be kept confidential. Indifference and lack of time were most frequently cited as reasons for not being willing to participate. Inclusion criteria were: (a) flight to Germany, (b) age 18 years or older, and (c) having given written informed consent. Exclusion criteria were suicidality, acute psychosis, or cognitive deficits. No participant had to be excluded for any of these reasons, but five participants were excluded due to missing data, resulting in a total of *N* = 99 (24).

Assessment took place from December 2017 to July 2018. Three master level psychology students conducted questionnaire-based semi-structured clinical interviews, with the support of altogether five interpreters (Arabic, Farsi, Kurdish) who were trained in translating in the refugee mental health context. The interviewers were supervised on a regular basis. On average, the interviews lasted 1 h. Participants received a voucher worth €10 as financial compensation. Participants who expressed high levels of distress during the interview were informed about counseling and treatment options. The study was performed in accordance with the ethical standards laid down in the 1964 Declaration of Helsinki and its later amendments. Ethical approval was granted by the Institutional Review Board of the Catholic University Eichstaett-Ingolstadt (approval number 2017/09).

Table 1 presents sociodemographic characteristics of the participants as well as information on traumatic and loss-related experiences [for more information see (24)], mental health symptoms, and homesickness. The total sample was aged 30 years on average, with a wide range from 19 to 74 years. More participants were male (68%) and the majority was from the Middle East (e.g., Syria and Iraq). On average, participants had arrived in Germany nearly a year and a half before, with a range from less than a month up to nearly 4 years. Nearly all participants (92%) reported the loss of at least one loved person (dead or gone missing), with 65% having experienced the loss of a nuclear family member, 90% the loss of an extended family member, and 89% the loss of a friend. Moreover, all participants had experienced at least one event that would be considered as potentially traumatic according to DSM-5 (28). Severe human suffering (75%), transportation accidents (74%), and combat or war-zone exposure (70%) were the most frequently reported events. Participants reported rather high levels of posttraumatic stress and depressive symptoms as well as symptoms of prolonged grief (see Table 1). As the analyses in Comtesse and Rosner (24) had shown, symptoms of posttraumatic stress and grief seemed to have diminished over time during the stay in Germany, while depressive symptoms were especially high in participants who had temporary resident permits and had stayed the longest in Germany.

**TABLE 1:** Participant characteristics.

Characteristic	Total (*N* = 99)
Gender, *n* (%)	
Male	68 (67.7)
Female	32 (32.2)
Age in years, *M* (*SD*)	30.12 (9.43)
Education in years, *M* (*SD*)	9.91 (4.79)
Ethnicity, *n* (%)	
Arabic	45 (45.4)
Kurdish	32 (32.3)
Afghan	15 (15.2)
Other	7 (7.1)
Time in Germany in months, *M* (*SD*)	16.56 (12.92)
Stage of asylum proceedings, *n* (%)	
Asylum request in process	29 (29.3)
In appeal against rejected asylum	32 (32.3)
Temporary residence permit	38 (38.4)
German proficiency, *M* (*SD*)^a^	6.00 (7.02)
Employment/vocational training, *n* (%)	48 (48.5)
Number of losses, *M* (*SD*)^b^	5.67 (6.47)
Number of traumatic events, *M* (*SD*)^c^	7.11 (3.53)
Homesickness (UHS), *M* (*SD*)	2.97 (0.80)
Factor 1: adjustment difficulties/loneliness^d^	1.91 (0.92)
Factor 2: ruminations about home^d^	1.45 (0.91)
Factor 3: missing family and/or friends^d^	2.07 (0.93)
Prolonged grief symptoms (TGI-SR), *M* (*SD*)	47.03 (20.05)
Posttraumatic stress symptoms (PCL-5), *M* (*SD*)	31.39 (18.33)
Depressive symptoms (PHQ-9), *M* (*SD*)	9.59 (6.74)

^a^Participants were asked for how many months they have been studying German.

^b^Includes family members or friends reported as missing.

^c^A sum score of the number of personally experienced and/or witnessed traumatic events was calculated.

^d^Scores are standardized (z-scores).

### Measures

Sociodemographic and migration-related information was obtained during the interview, as well as information regarding asylum proceedings and successful integration, for example, work status and proficiency in German.

The *Utrecht Homesickness Scale* (UHS) (27) was used to assess homesickness experienced during the past 4 weeks on a 5-point scale (1 = *not at all*, 5 = *very strongly).* The 20-item UHS comprises five dimensions (missing family, missing friends, loneliness, ruminations about home, adjustment difficulties), each assessed with four items. The reported Cronbach’s alphas for the five subscales were high, ranging from 0.80 to 0.90, as was the alpha for the total UHS score, 0.94 (29). The respective five-factor structure has been confirmed in a sample of university students (30). However, Stroebe et al. (17) suggested analyzing home-focused items (e.g., “Missing your family”) separately from items referring to adjustment to the new environment (e.g., “Having difficulties in getting used to new customs”) because separation distress and adjustment problems are different sources of distress when coping with relocation.

Mental health was assessed via the following self-report measures [see (24), for a more detailed description]: The 9-item *Patient Health Questionnaire* [PHQ-9; (31)] for assessing depressive symptom severity according to the DSM-IV (9 items on a scale from 0 to 3); the *PTSD Checklist for DSM-5* [PCL-5; (32)] for assessing posttraumatic stress symptoms (20 items on a scale from 0 to 4), together with the 17-item *Life Events Checklist for DSM-5* [LEC-5; (32)] for assessing traumatic events; the 18-item *Traumatic Grief Inventory Self-Report Version* [TGI-SR; (33)] for assessing prolonged grief symptom severity (scale ranging from 1 to 5). Interpersonal loss exposure was assessed with questions of how many cases of death or cases of loved ones gone missing have occurred within the nuclear family (spouse, child, parent, sibling), among other relatives, and among close friends. These types of losses (i.e., death or missing loved ones) were added up to create indices of the total number of losses.

### Data analysis

To explore the dimensionality of homesickness as assessed with the UHS (27), we computed an iterated principal factor analysis with promax rotation [see (34), for example]. Exploratory factor analysis was chosen as procedure as the sample was not large enough for confirmatory factor analysis and the UHS was employed for the first time in refugees. Factors were extracted on the basis of Horn’s parallel analysis (35) and the screen test (36). We extracted three factors (see “Results” section and Table 2). Individual factor scores were calculated with the regression method (37) and fed into all subsequent analyses. In addition, analyses were performed with the total score of the UHS, amounting to four homesickness scores altogether.

**TABLE 2:** Utrecht Homesickness Scale (UHS): Iterated principal factor analysis with promax rotation.

	Factor		
	(1)	(2)	(3)
Eigenvalues	5.37	2.18	1.10
Percentage of explained variance	54%	22%	11%
**UHS Item**	**Factor loadings**		
(1) **Adjustment difficulties/loneliness**			
5. Feeling lonely	**0.68**	−0.05	−0.01
7. Feeling uprooted	**0.63**	0.18	0.01
3. Feeling lost in the new situation	**0.60**	0.07	0.18
4. Feeling isolated from the rest of the world	**0.58**	0.07	−0.09
15. Having difficulties in getting used to new customs	**0.58**	−0.05	0.15
1. Finding it difficult adjusting to a new situation	**0.56**	0.25	0.01
2. Feeling uncomfortable in a new situation	**0.49**	0.02	0.12
6. Feeling unloved	**0.37**	−0.01	−0.22
19. Repeatedly thinking of the past	**0.30**	0.25	0.24
(2) **Ruminations about home**			
16. Continuously having thoughts about home	0.01	**0.72**	−0.02
18. Having thoughts that an old situation was better than here and now	0.27	**0.60**	−0.07
11. Missing home	0.16	**0.58**	0.07
17. Regretting the decision to leave an old situation	**0.35**	**0.49**	0.01
8. Missing people whom you trust and can talk with	0.01	**0.40**	**0.37**
10. Searching for familiar faces	−0.01	**0.36**	−0.04
(3) **Missing family and/or friends**			
14. Missing your parents	0.11	−0.12	**0.85**
20. Feeling missed by your family	0.09	0.03	**0.75**
12. Missing your family	−0.02	0.07	**0.72**
13. Missing your friends	−0.05	**0.45**	**0.46**
9. Longing for acquaintances	−0.17	**0.35**	**0.39**

*N* = 99.

Factor loadings from the pattern matrix ≥ | 0.30| are in boldface. Items were asked in Arabic, Farsi, or Kurdish. UHS, Utrecht Homesickness Scale.

To explore possible predictors of homesickness, we first calculated Spearman’s rank correlations between homesickness scores and socio-demographic (gender, age, education, employment/vocational training, and proficiency in German) as well as stressor- (number of losses, traumatic events) and migration-related variables (time since arrival in Germany, stage of the asylum proceedings) and current psychological symptoms (regarding PGD, PTSD, and depression). Variables that showed significant correlations with at least one of the homesickness scores were included in a multiple regression analysis for each of the four scores. All tests were two-tailed with α = 0.05 without correcting for multiple comparisons as this research was exploratory in nature. Analyses were conducted using Stata version 15.0 (StataCorp, USA).

## Results

### Homesickness in asylum seekers as assessed by the Utrecht Homesickness Scale

The total homesickness score in our sample was *M* = 2.97 (*SD* = 0.80, range: 1.40–4.71), being somewhat higher than in most samples of university students that ranged from 1.93 to 2.43 (29, 38) and in expatriate employees with an UHS total of 1.76 [*SD* = 0.54; (27)]. Cronbach’s alpha of the total UHS scale was good, with α = 0.86.

Exploratory factor analysis did not yield a five-factor structure as in Stroebe et al. (29), with only three factors having Eigenvalues ≥ 1 (see Table 2) and Horn’s parallel analysis strongly suggesting three factors. Inspection of the scree plot suggested either a one or a three-factor solution. We therefore extracted three factors that accounted for 87% of the total variance (see Table 2). Based on item loadings, Factor 1 could be interpreted as “Adjustment difficulties/loneliness,” Factor 2 as “Ruminations about home,” and Factor 3 as “Missing family and/or friends.” With one exception (Item 19), the first factor perfectly combined the two respective factors “loneliness” and “adjustment difficulties” found by Stroebe et al. (29). Factor 2, however, was only loosely equivalent to the original factor “ruminations about home,” and while Factor 3 contained three items of Stroebe et al.’s original factor “missing family,” the fourth item of the original subscale (Item 11, “missing home”) clearly loaded on Factor 2. Also, Factor 3 included two items about missing friends which was a distinct factor in Stroebe et al., so, Factor 3 was more generally about missing loved ones. All in all, the factor structure and item loadings in our sample deviated substantially from the original five-factor structure. Therefore, we computed individual factor scores for each of the three factors derived in this study.

### Predictors of homesickness in multiple regression analysis

First, to identify the most relevant variables that should be included in the regressions, we computed Spearman’s rank correlations (see Table 3). Mean factor scores of the three homesickness factors were correlated positively but not intercorrelated completely, as the Adjustment factor score did not correlate significantly with the Missing factor score, supporting the procedure of computing distinct regression analyses for each mean factor score instead of only for the UHS total score.

**TABLE 3:** Spearman’s rank correlations between homesickness scores and possible predictors.

Variable	1	2	3	4	5	6	7	8	9	10	11	12	13	14	15	16
1. UHS (total)	−															
2. Adjustment difficulties/loneliness	0.77***	−														
3. Ruminations about home	0.78***	0.34***	−													
4. Missing family and/or friends	0.61***	0.17	0.41***	−												
5. Gender^a^	–0.06	–0.02	0.03	−0.22*	−											
6. Age	0.06	–0.12	0.21*	0.05	0.02	−										
7. Education (in years)	–0.03	–0.05	0.02	0.00	–0.04	0.09	−									
8. German proficiency^b^	0.06	0.12	0.14	–0.19	–0.12	–0.10	0.19	−								
9. Employment/vocational training^c^	0.18	0.09	0.18	0.16	–0.20	0.00	0.12	0.45***	−							
10. Time in Germany	0.19	0.31**	0.21*	–0.18	–0.03	0.12	–0.04	0.61***	0.39***	−						
11. Stage of asylum proceedings^d^	0.25*	0.09	0.46***	–0.01	0.03	0.24*	0.14	0.52***	0.40***	0.56***	−					
12. Number of losses^e^	0.24*	0.02	0.43***	0.08	–0.05	0.48***	0.06	0.17	0.15	0.31**	0.49***	−				
13. Number of traumatic events^f^	0.09	0.16	0.05	–0.09	−0.35***	0.05	0.06	0.16	0.00	0.19	0.02	0.27**	−			
14. Prolonged grief symptoms (TGI-SR)	0.13	0.12	–0.11	0.25*	–0.01	–0.06	0.08	−0.26*	–0.11	−0.32**	−0.33**	–0.02	–0.04	−		
15. Posttraumatic stress symptoms (PCL-5)	0.26*	0.31**	0.00	0.11	–0.02	–0.15	–0.20	−0.21*	–0.21	−0.22*	−0.36***	–0.06	0.16	0.47***	−	
16. Depressive symptoms (PHQ-9)	0.35***	0.40***	0.23*	0.00	0.13	0.10	–0.01	0.21*	0.10	0.22*	0.22*	0.15	0.17	0.11	0.24*	−

UHS, Utrecht Homesickness Scale; TGI-SR, Traumatic Grief Inventory Self-Report Version; PCL-5, PTSD Checklist for DSM-5; PHQ-9, Patient Health Questionnaire.

^a^Coded as 0 “male,” 1 “female”; ^b^Participants were asked for how many months they have been studying German; ^c^Coded as 0 “none,” 1 “employment/vocational training”; ^d^Coded as 0 “uncertain” (i.e., asylum request in process or in appeal against rejected asylum), 1 “certain” (i.e., temporary residence permit); ^e^Includes family members or friends reported as missing. ^f^A sum score of the number of personally experienced and/or witnessed traumatic events was calculated.

***p < 0.001, **p < 0.01, *p < 0.05.

In a second step, the following variables were included as possible predictors in multiple linear regression analyses, based on at least one significant correlation with one of the homesickness scores: gender, age, time since arrival in Germany, stage of asylum proceedings (dichotomized), number of losses, depressive symptoms, posttraumatic stress symptoms, and prolonged grief symptoms. In Table 4, results of the four multiple linear regressions on the UHS total score and the three factor scores are presented. For UHS total, and for the “Adjustment difficulties/loneliness” and “Ruminations about home” factor scores, the overall models were significant, explaining from 19 to 26% (adj. *R*^2^) of the respective homesickness score’s variance. The regression model for the “Missing family/and or friends” factor scores was not significant, although reporting prolonged grief symptoms and being male was associated in bivariate correlations with missing friends and family (see Table 3). The variance inflation factor (VIF) did not indicate serious multicollinearity among potential predictor variables (all VIFs were < 2.13).

**TABLE 4:** Multiple regression analyses to predict homesickness (UHS total and factor scores).

	UHS total			(1) Adjustment difficulties/loneliness			(2) Ruminations about home			(3) Missing family and/or friends		
				
Predictor	*B (SE)*	β	*p*	*B (SE)*	β	*p*	*B (SE)*	β	*p*	*B (SE)*	β	*p*
Gender^a^	–0.14 (0.15)	–0.08	0.354	–0.09 (0.17)	–0.04	0.592	0.04 (0.17)	0.02	0.820	–0.45 (0.19)	–0.22	**0.023**
Age	–0.01 (0.01)	–0.03	0.713	–0.01 (0.01)	–0.11	0.254	0.00 (0.01)	0.01	0.902	0.00 (0.01)	0.03	0.743
Time in Germany	0.01 (0.01)	0.05	0.636	0.02 (0.01)	0.34	**0.002**	–0.01 (0.01)	–0.10	0.341	–0.02 (0.01)	–0.23	0.051
Stage of asylum proceedings^b^	0.43 (0.21)	0.26	0.051	0.05 (0.23)	0.03	0.815	0.75 (0.23)	0.40	**0.002**	0.33 (0.27)	0.17	0.215
Number of losses^c^	0.01 (0.01)	0.09	0.409	–0.01 (0.02)	–0.06	0.548	0.03 (0.01)	0.25	**0.030**	0.01 (0.02)	0.04	0.725
Grief symptoms (TGI-SR)	0.01 (0.00)	0.08	0.415	0.00 (0.00)	0.07	0.479	–0.01 (0.00)	–0.06	0.507	0.01 (0.01)	0.20	0.074
Posttraumatic stress symptoms (PCL-5)	0.01 (0.01)	0.27	**0.018**	0.01 (0.01)	0.26	**0.013**	0.01 (0.01)	0.15	0.164	0.00 (0.01)	0.03	0.758
Depression (PHQ-9)	0.03 (0.01)	0.21	**0.040**	0.04 (0.01)	0.28	**0.005**	0.01 (0.01)	0.08	0.372	0.00 (0.01)	0.01	0.973
adjusted *R*^2^/*F*	0.19/3.85***			0.26/5.39***			0.24/4.95***			0.07/1.96		

Values in bold refer to significant *p* values.

UHS, Utrecht Homesickness Scale; TGI-SR, Traumatic Grief Inventory Self-Report Version; PCL-5, PTSD Checklist for DSM-5; PHQ-9, Patient Health Questionnaire. ^a^Coded as 0 “male,” 1 “female.” ^b^Coded as 0 “uncertain” (i.e., asylum request in process or in appeal against rejected asylum proceedings), 1 “certain” (i.e., temporary residence permit); ^c^Includes family members or friends reported as missing.

***p < 0.001.

The UHS total score was significantly associated with depressive and posttraumatic stress symptom severity. This was also the case regarding the “Adjustment difficulties/loneliness” factor scores, but here, time since arrival in Germany emerged as the strongest predictor: The longer individuals had been staying in Germany, the more adjustment difficulties and loneliness they reported. Higher scores regarding the Rumination factor were not associated with mental health symptoms but with a higher number of losses and the stage of the asylum proceedings. Here, the dichotomized variable for asylum status emerged as the most important predictor, insofar that individuals who had been granted a temporary residence permit had higher factors scores regarding “Ruminations about home” as those whose asylum request was either still in process or who had appealed against a rejected asylum plea.

## Discussion

In a sample of 99 individuals who had sought asylum in Germany and were living in collective accommodation facilities for nearly one and a half years on average, participants showed a pronounced level of homesickness, assessed with the UHS (29, 27). So far, the UHS had only been used to measure homesickness in samples of students who had left home for studying at the university [e.g., (29, 38)] or in employees who were posted abroad (27). Our aim in this secondary analysis of data on the mental health of asylum seeking individuals in Germany (24) was to explore the usability of the UHS for assessing homesickness in a refugee population and to shed more light on this construct and its association with mental health and migration-related variables. In our sample, the UHS was easy to apply and reliable, with a good Cronbach’s alpha (α = 0.86) of the total UHS scale. Instead of the original five-factor structure reported in Stroebe et al. (29), the best solution were three factors explaining 87% of the total variance. In fact, the first factor in our solution “Adjustment difficulties/loneliness” corresponded well with the two distinct factors “loneliness” and “adjustment difficulties” found in Stroebe et al. (29). However, the two other factors in our solution, “Ruminations about home” and “Missing family and/or friends” were less comparable to Stroebe et al.’s respective factors, with some items loading differently and the Missing factor combining items on missing family as well as on missing friends. Still, our results fit with Stroebe et al.’s (17, 20) suggestion of differentiating between what they coined a “new place factor” (tapping relocation phenomena and adjustment difficulties—and in our sample feelings of loneliness and isolation as well) and a “home factor” which is about missing one’s home and loved ones. These two aspects—the “new place” and the “home” factor—emerged as quite distinct, as Factor 1 “Adjustment difficulties/loneliness” and Factor 3 “Missing family and/or friends” did not correlate significantly. Factor 2 “Ruminations about home,” however, was somewhere in between, associated both with Factors 1 and 3.

More importantly, different variables emerged as significant predictors of homesickness factors. While higher mean factor scores for “Adjustment difficulties/loneliness” were associated with reporting more depressive and posttraumatic symptoms but also with having stayed longer in the host country, “Ruminations about home” factor scores were solely associated with migration-related variables, namely number of losses and—emerging as strongest predictor—the stage of asylum proceedings, with those asylum seekers who were granted a temporary resident permit having higher scores regarding Factor 2. Looking at bivariate correlations, both time since arrival and stage of asylum proceedings were significantly associated with ruminating about home. Having finally achieved a residence permit, albeit temporary, might be associated with finding more mental capacities to focusing on what has been lost. This part of the overall construct of homesickness as measured with the UHS might be related to what Eisenbruch (7) coined “cultural bereavement,” which he defined resulting “from the loss of social structures, cultural values and self-identity,” leading individuals to live in the past and suffering from “feelings of guilt over abandoning culture and homeland” (p. 674). It also reflects what once was called “nostalgia,” which is literally “longing for home so much that it hurts.” Accompanying this hurt is a tendency to idealize what has been left behind. Idealizing might be easier the longer separation continues. Finally, the variables we chose for our regressions did not sufficiently explain enough variance of the “Missing family and/or friends” factor scores for the overall prediction model to reach significance. This is surprising as one might expect a more close relationship with prolonged grief symptoms, which were significantly related to the missing factor in bivariate correlations, in the sense that reporting more prolonged grief symptoms went with higher scores regarding missing family and/or friends. In the light of the rather small sample size we cannot rule out a lack of power in the analysis that prevented results to reach significance. It is noteworthy, however, that “Missing family and/or friends” did not correlate at all with depressive or posttraumatic stress symptoms, while studies that looked at the impact of actual separation from family members found a negative effect on mental health (e.g., 11, 13, 14).

Taken together, reporting more overall homesickness was associated with more severe mental health issues, especially with depressive symptoms—and in our sample of asylum seekers also with posttraumatic stress symptoms. The relationship between depression and homesickness is in line with the overall research on homesickness in other populations [e.g., (19); for an overview see (15)]. However, in our sample, the association with mental health symptoms was mainly driven by either those items that tapped adjustment problems regarding the new situation (e.g., “Having difficulties in getting used to new customs”) or those that tapped feelings of isolation (e.g., “Feeling lonely”), that is, Factor 1 (see Table 2 for more examples). So, it could be useful to differentiate between an adjustment-related “new place factor” and the (missing/longing for) “home factor” (17), as adjustment-related problems and loneliness seem to be associated more strongly with depressive symptoms than missing family or friends. In that respect, our results do match with results from Stroebe et al. (29), but when it comes to the role of time, our results do not fit with those found in students, where homesickness grew less intense over time. In fact, in the context of forced migration where lengthy asylum proceedings and other ongoing post-migration stressors like economic hardships worsen mental health outcomes [e.g., (22)], it seems not that surprising that time since arrival in Germany emerged as important predictor specifically for “Adjustment difficulties/loneliness” factor scores in cross-sectional analysis, as this factor was also related to depressive and posttraumatic stress symptoms. However, research on the “time variable” as predictor of mental health symptoms in refugee populations showed mixed results leading to the assumption that its role depends on context and that it probably should be considered as a marker for other ongoing post-migration stressors (39). For example, in a recent systematic review on the role of post-migration stressors in refugees and asylum seekers in Germany, housing quality emerged as an important factor, with living in collective accommodation (as opposed to private accommodation) being related to worse mental health (40). As we had recruited study participants exclusively in collective accommodation facilities—and most of them resided in these kind of accommodation for more than a year—adjustment difficulties might have worsen over time.

### Strengths and limitations

As far as we know, this is the first study assessing homesickness in asylum seeking individuals with more than one or just a few items as included in instruments measuring post-migration or acculturative stress, for example the *Demands of Immigration Scale* [DIS; (41)]. Instead, we used a 20-item instrument explicitly developed to measure homesickness. Another strength of this research is that we conducted the assessment with semi-structured interviews that allowed individuals to ask if they had problems with understanding a question, which is important with participants from diverse cultural backgrounds and with different levels of literacy. Assessment was conducted in three different languages (Arabic, Farsi, and Kurdish) with the help of interpreters who were trained in translating in the refugee mental health context. Still, we cannot rule out the possibility that semantic differences or differences in understanding occurred in our ethnically diverse sample, as the three versions of the UHS were not translated back and forth to better validate the translations. Also, the small sample size did not allow for comparisons between different ethnic backgrounds or languages spoken. As we could not replicate the original five-factor structure reported by Stroebe et al. (29), we computed factor scores for further analysis instead of computing subscales according to our three-factor solution—which we actually deem as a strength in light of the exploratory nature of researching homesickness in refugees and asylum seekers. Again, the small sample size is a limitation and the factor structure of the UHS should be evaluated in a larger sample of refugees, for further validation. Finally and in a similar vein, we should have included more post-migration factors that are known to be related to refugees’ adjustment and mental health in their host country (22, 42). To better differentiate genuine homesickness and nostalgia (“home factor”), which might be captured more by the items that loaded on our Factors 2 and 3 (ruminations about home and missing family and/or friends) from adjustment problems (“new place”), more post-migration variables could be taken into account. In particular, actual family separation should be assessed, although from what we learned up to now, we would be hesitant to predict a simple linear association in the sense of “more separation, more homesickness.” Research on this topic is still in its early, exploratory phase.

### Conclusion and clinical implications

Our study not only shows that it is feasible to assess homesickness in a refugee population with the UHS, but also suggests that it might be clinically worthwhile: Homesickness as measured with the UHS total was associated with more depressive and posttraumatic stress symptoms. Differentiating between an adjustment-related “new place factor” and the “home factor” that addresses the aspects and consequences of separation from home and loved ones (17) seems useful as especially problems adjusting to the new situation seem to promote the association with more severe mental health issues in refugees. One might argue that the correlation between depressive symptoms and our Factor 1 “Adjustment difficulties/loneliness” could be an artifact, caused by the items that tap feelings of loneliness. However, there is less conceptual overlap with posttraumatic stress symptoms, which were associated with Factor 1 as well. Also, if there’s some overlap, this calls even more for looking separately at the different sides of feeling homesick as reported in the UHS. In any case, our sample showed a pronounced amount of homesickness, which could be explained by several reasons: As a rule, they did not chose to be separated in the first place (in contrast to students, for example) but dire circumstances forced them to leave home and loved ones. In addition, temporal separation often mingles with perpetual loss—be it perpetual loss of home (because it was destroyed or for political reasons) or of loved ones (because they were killed or are missing). Finally, refugees are often confronted with very demanding “new place” stressors, for example, very low or no income, restrictions concerning housing or employment, and uncertain asylum status; these are all social determinants of mental health, clinicians should be aware of (22). Therefore, addressing both sides of the coin makes clinical sense. Following their earlier model for adaptive coping with bereavement (43), Stroebe et al. (20) propose a dual process model of coping with homesickness (DPM-HS) that might inform clinicians working with refugees as well: Suffering from homesickness, individuals oscillate between intrusion and avoidance concerning both loss-related symptoms and adjustment problems; they need to acquire more functional emotion regulation strategies that allow them to cope with grief and yearning as well as with everyday demands. From our clinical experience, homesickness is a recurring theme that is brought up often in treatment by patients who were forced to leave their country and who are treated for PTSD and/or other mental health problems. However, the data from this study are not sufficient to conclude whether homesickness could be considered as risk factor aggravating the mental health of refugees or rather as a consequence of poor psychological adjustment. Also, they do not suggest a close relationship between homesickness and prolonged grief symptoms (i.e., more pathological forms of grieving). Future research should be longitudinal and take a closer look to the role of homesickness *per se* and in combination with adjustment problems and acculturative and other post-migration stressors for the course of mental health symptoms. In the context of forced migration, treatment research in particular might gain from taking homesickness into account.

## Data availability statement

The data that support the findings of this study will be made available by the last author, HC, hannah.comtesse@ku.de, without undue reservation.

## Ethics statement

The studies involving human participants were reviewed and approved by the Institutional Review Board of the Catholic University Eichstaett-Ingolstadt. The participants provided their written informed consent to participate in this study.

## Author contributions

RR and HC designed the study. HC obtained the funding. RR and MH wrote the first draft. HC computed the analyses. LB and HC edited and commented on subsequent manuscript versions. All authors read and approved the final version of the manuscript.
